# Temperature management during cytoreductive surgery with hyperthermic intraperitoneal chemotherapy

**DOI:** 10.3389/fonc.2022.1062158

**Published:** 2023-01-19

**Authors:** Maria F. Ramirez, Juan Jose Guerra-Londono, Pascal Owusu-Agyemang, Keith Fournier, Carlos E. Guerra-Londono

**Affiliations:** ^1^ Department of Anesthesiology and Perioperative Medicine, The University of Texas MD Anderson Cancer Center, Houston, TX, United States; ^2^ Department of Surgical Oncology, The University of Texas MD Anderson Cancer Center, Houston, TX, United States; ^3^ Department of Anesthesiology, Pain Management, & Perioperative Medicine, Henry Ford Health System, Detroit, MI, United States

**Keywords:** HIPEC, hyperthermia, temperature control, cooling protocols, chemotherapy, peritoneal disease

## Abstract

In addition to attaining complete or near complete cytoreduction, the instillation of select heated chemotherapeutic agents into the abdominal cavity has offered a chance for cure or longer survival inpatients with peritoneal surface malignancies. While the heating of chemotherapeutic agents enhances cytotoxicity, the resulting systemic hyperthermia has been associated with an increased risk of severe hyperthermia and its associated complications. Factors that have been associated with an increased risk of severe hyperthermia include intraoperative blood transfusions and longer perfusion duration. However, the development of severe hyperthermia still remains largely unpredictable. Thus, at several institutions, cooling protocols are employed during cytoreductive surgery with hyperthermic intraperitoneal chemotherapy (CRS-HIPEC). Cooling protocols for CRS-HIPEC are not standardized and may be associated with episodes of severe hyperthermia or alternatively hypothermia. In theory, excessive cooling could result in a decreased effectiveness of the intraperitoneal chemotherapeutic agents. This presumption has been supported by a recent study of 214 adults undergoing CRS-HIPEC, where failure to attain a temperature of 38° C at the end of chemo-perfusion was associated with worse survival. Although not statistically significant, failure to maintain a temperature of 38° C for at least 30 minutes was associated with worse survival. Although studies are limited in this regard, the importance of maintaining a steady state of temperature during the hyperthermic phase of intraperitoneal chemotherapy administration cannot be disregarded. The following article describes the processes and physiological mechanisms responsible for hyperthermia during CRS-HIPEC. The challenges associated with temperature management during CRS-HIPEC and methods to avoid severe hypothermia and hyperthermia are also described.

## Introduction

Peritoneal dissemination of disease is a common manifestation of gastrointestinal and gynecological malignancies including those of ovarian, colon, gastric, small intestine and appendiceal origin (1). Among the peritoneal surface malignancies, disease of colorectal origin is most common with an estimated prevalence of about 5% (2).

According to a recent study published in the Journal of the American Medical Association, approximately 60,000 patients are diagnosed with peritoneal disease in the United States every year (3). However, over the last couple of decades, there has been an increase in the incidence of the disease, which can be explained by the improvement and accessibility to diagnostic imaging (computed tomography and ultrasound) and the introduction of screening colonoscopy for high-risk patients (4).

The presence of peritoneal disease is associated with more rapid disease progression, poor prognosis, and a significant decrease in survival. As expected, survival rates differ according to the location and histology of the primary tumor. For instance, according to the multicentric prospective study EVOCAPE I (Evolution of Peritoneal Carcinomatosis), the median survival in patients with peritoneal disease is 2.1 months for those with pancreatic cancer, 5.2 months for advanced colorectal cancer and 3.1 months in patients with advanced gastric cancer (5).

Despite significant advances in treatment, systemic chemotherapy alone has shown to have minimal effect on the progression of certain types of peritoneal disease (6). Furthermore, systemic chemotherapy is often associated with severe dose-limiting toxicity in many patients and as a result cytoreductive surgery combined with hyperthermic intraperitoneal chemotherapy (CRS-HIPEC) has become a more popular treatment. CRS-HIPEC offers an opportunity for the eradication of macroscopic disease and treatment of microscopic disease, with a benefit of a decreased risk of systemic toxicity and prolongation of survival.

CRS-HIPEC is an extensive surgical procedure that has become part of the standard of care for patients with a select group of peritoneal surface malignancies (7). The procedure typically involves multiple organ resections, peritonectomies, and the instillation of heated chemotherapy (up to 42o C) into the abdominal cavity for up to 120 minutes. The goal of intra-abdominal hyperthermia during CRS-HIPEC is to enhance the cytotoxicity and penetration of chemotherapeutic agents into malignant disease (8, 9). The mechanism for this synergistic effect may be related to 1) hyperthermia-induced increased permeability of chemotherapeutic agents into tumor cells, 2) increased drug-induced DNA damage, 3) inhibition of the repair of drug-induced DNA damage, 4) and the expression of heat shock proteins by tumor cells which ultimately potentiates the effect of Natural Killer cells (antitumor response) (10, 11).

## HIPEC technique

**Figure 1:**
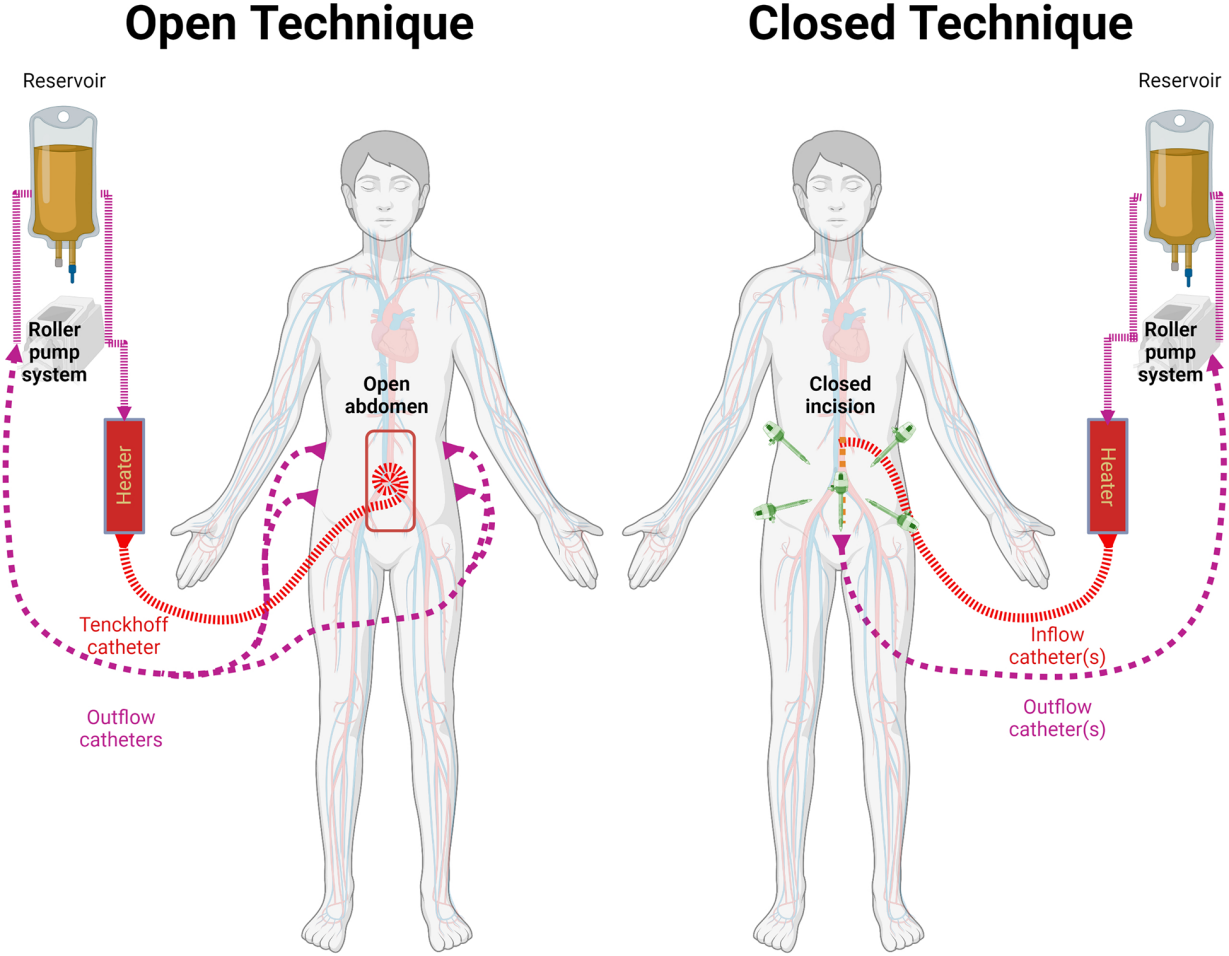
Methods for HIPEC administration. Both methods involve the use of a reservoir, roller pump system, heater, and connecting circuits. Temperature is monitored through the HIPEC procedure. Left: the open technique keeps the cavity open and retracts the wall to increase its filling capacity.Typically uses a Tenckhoff catheter for inflow and 2 catheters on each side for outflow as described by Sugarbaker et al. Right: the cavity is closed temporarily or definitely for HIPEC. Variable number of cannulas are used. In recent years it has been used after laparoscopic cytoreductive approach.

HIPEC is typically delivered to the patient in the operating room after cytoreduction surgery has been completed and hemostasis is confirmed. The procedure involves placement of cannulas that introduce (inflow) and remove (outflow) fluid from the abdominal cavity, which is then recirculated through a perfusion circuit driven by a roller pump. To ensure adequate flow between the inflow and the outflow cannulas, the peritoneal cavity it filled with fluid (filling phase) and the perfusion machine adjusted to keep a steady flow between the reservoir and the patient (12). Occasionally, the perfusionist may need to add fluid to the circuit in order to achieve this goal. The heat exchanger keeps the perfusate temperature at 43 to 45°C. The goal of this is to maintain the intraperitoneal temperature between 41 and 43°C. Once the in/out flow and temperatures are relatively stable, the chemotherapeutic agent is added to the pump primer (12, 13). This solution, also known as the perfusate, is circulated between the patient and the machine for up to 120 minutes. During this time, temperature probes within the abdominal cavity provide information regarding the degree of hyperthermia. There are two methods to administer HIPEC (**Figure 1**): the open coliseum technique and the closed abdomen technique (13–17). During the open technique, the abdominal wall skin edges are elevated with a retractor and the abdominal contents are directly agitated manually. In contrast, in the more popular closed technique, the skin is completely sutured closed along the laparotomy incision and the abdominal wall is manually agitated during the perfusion time to promote uniform heat distribution throughout the peritoneal cavity.

Although each technique possesses unique advantages (**Table 1**), such as the capacity to manually stir the fluid in the open technique or the ability to rapidly achieve and maintain hyperthermia in the closed technique, neither has demonstrated superior outcomes compared to the other (12, 14, 18). In recent years, following the increasing use of minimally invasive surgery, some patients have received laparoscopic HIPEC, avoiding the need of a midline laparotomy to place the cannulas by using the initial laparoscopy ports (7, 24–26).

**Table 1:** Comparison of open and closed HIPEC techniques.

Features	Closed technique	Open technique
**Technology variations**	May be used with minimally invasive cytoreduction.	Traditional open coliseum, “closed technique” with open access (7).
**Temperature control**	Easier to achieve target temperature (18).	More difficult to reach target temperature (18).
**Chemotherapy distribution**	Dependent on abdominal distention, pressure, and external shaking. Pooling of chemotherapy is conceivable (18).	Manual stirring of fluid and organs is possible (7).
**Temperature distribution**	Follows the inflow-to-outflow gradient (at studied parameters) (19).	Manual stirring of fluid and organs is possible. Heat loss dissipates over the exposed abdomen (additional posterior-to-anterior gradient).
**Volume**	Limited to usual filling capacity of the cavity. Smaller variations in total volume used between studies (20).	Increased due to tenting of the abdominal wall. Larger variations in total volume used between studies (20).
**Pressure**	Can be increased. May improve tissue penetration (21, 22).	No additional pressure can be exerted.
**Occupational hazard**	Closed circuit limits agent exposure. Risk of a splash accident could still occur.	Room staff at higher risk of splash and aerosolization. Surgeon may decrease skin exposure with double gloving (23).
**Visualization of cavity**	Only performed at the end of perfusion. A laparoscopic HIPEC alternative for real-time assessment has been described (26917929)	Allows detection of immediate complications and continued cytoreduction (7).
**Physiologic Changes**	Related to core-body hyperthermia.	Related to core-body hyperthermia and intrabdominal pressure.

The chemotherapeutic agents administered during HIPEC include cisplatin, oxaliplatin, mitomycin C, paclitaxel, and doxorubicin. These chemotherapeutic agents are employed during HIPEC procedures since they are stable at high temperatures and have a synergic effect with heat (8). Noticeably, the cytotoxic effect of intraperitoneal chemotherapy depends on the concentration of the drug and the duration of chemotherapy instillation. The former, depends on the pharmacokinetic properties of the drug (e.g., half-life), the type of fluid administered along with chemotherapy (isotonic saline or dextrose containing solution), and the volume infused (7).

The most common core target temperature during HIPEC is 42°C. However measurements at different sites in the abdomen can be highly variable (20). For instance, inflow temperatures in recent trials have ranged from 41 to 45°C, while aiming for target intra-abdominal fluid temperatures between 40 and 43°C (27–29).

HIPEC machines available are either custom-made commercial devices (e.g., ThermoChemTM, Hyperthermia PumpTM, PerformerHTTM), or ‘homemade’ devices (cardiopulmonary bypass machine used in conjunction with a water bath) (7, 30). Some commercial machines heat the solution through a water bath, while others use electromagnetic induction. As mentioned before, all devices have a reservoir that helps adjust the fluid volume to the peritoneal cavity, compensates for variable outflow volume, prevents the circulation of air, and quickly removes the solution from the abdomen in case of emergency (12). Anecdotally, the volume of this reservoir is typically maintained at around 500 mL. Although one inflow and one outflow line are always connected to the HIPEC machine, there is a variable number of cannulas or catheters that reach the patient. According to Gronau et al., these numbers are seldom reported (31). Given that the volume of the solution held between the reservoir and circuit is variable or sometimes even unknown, the actual amount of chemotherapy in contact with the patient at a given time depends entirely on the individual HIPEC set up.

A novel approach to intraperitoneal chemotherapy instillation using pressurized aerosolized chemotherapy (PIPAC) has been described and tested in humans (32, 33). PIPAC aims to address the shortcomings of HIPEC by improving the distribution and penetration of chemotherapy and by reducing local and systemic toxicities (33). Additionally, PIPAC allows the precise determination of instant and total drug given (32). Further technological advances have also combined PIPAC with therapeutic hyperthermia (hPIPAC) (34). Proponents of this technique describe that the drug regimens used in PIPAC are more stable than those used for HIPEC (35). Currently, PIPAC is mostly perceived as a palliative or neoadjuvant therapy (in preparation for CRS/HIPEC). (PMID: 35602919. As PIPAC is not routinely administered with hyperthermia, further discussion is out of the scope of this manuscript.

## Biophysical considerations of intra-abdominal hyperthermia

In order to understand the temperature changes during CRS-HIPEC, it is necessary to comprehend principles of physics and human thermoregulation. From the perspective of physics, one can describe the human body (bounded by the skin) as an open thermodynamic system. During HIPEC, this system is surrounded by the operating room, the operating table, the perfusion machine, and the cooling systems. Altogether, these comprise the thermodynamic universe in which we observe the flow of energy. In this context, a HIPEC is “simply” the flow of thermal energy through the body over a predetermined time.

Because only one inflow and one outflow line are connected to the HIPEC machine, the energy transmitted to the patient can be calculated by using; 1) the difference between the inflow and outflow temperatures, 2) the HIPEC flow, and 3) the specific heat capacity of the hyperthermic fluid. For distilled water and normal saline, the specific heat capacities have been estimated to be 4179 and 4139 J Kg-1°C-1, respectively (36). This energy results in a temperature change consistent with the heating properties of the tissues (37).

**Figure 2:**
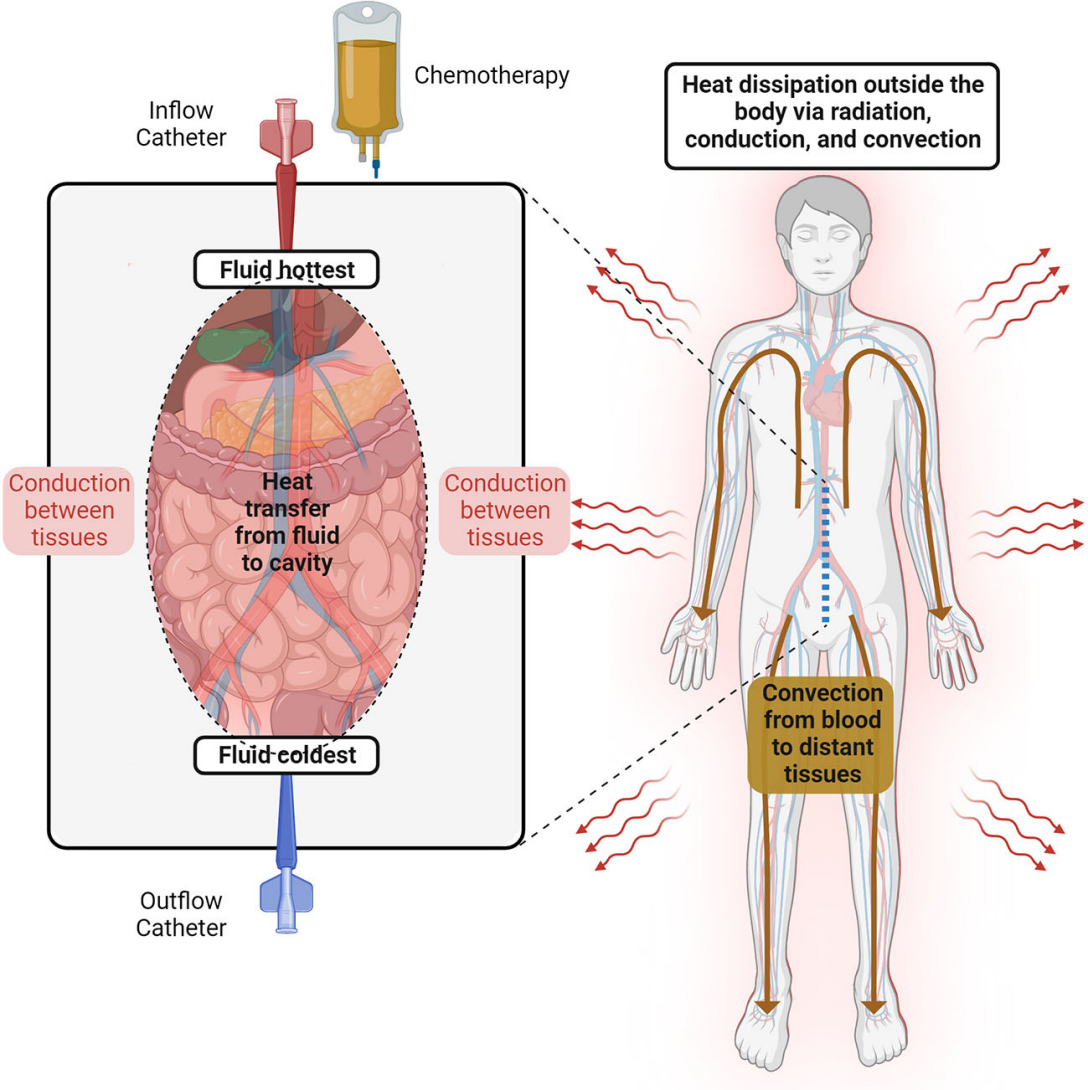
Temperature behavior during HIPEC. The heated perfusate is recirculated through the abdominal cavity. The fluid temperature follows a gradient between the inflow and the outflow catheters. Inside the cavity, the heat is dissipated between adjacent tissue layers via conduction, while distant tissues receive heat via convection from the blood flow. Thermoregulatory responses to hyperthermia (e.g., vasodilatation and sweating) allow heat dissipation outside the human body.

In general, heat transfer is the result of the balance between heat gain and heat loss. Heat gain is defined by the basal metabolic rate of the patient and the hyperthermic fluid, whereas heat loss is the result of the body’s interaction with the colder surroundings, such as the cooling systems, clothing, and the operating room. Over the last few decades, extensive research in thermal engineering has improved our knowledge of the human thermal responses to different environmental conditions, which has resulted in multiple predictive thermophysical and mathematical models (38–40). As explained by Stolwijk, a human thermophysical model consists of a *passive* (*controlled*) and an *active (controlling)* system. The *passive* system is composed of the tissues (and their respective heating properties) and the circulatory system (41). Within the body, heat is transferred by conduction between adjacent tissue layers and by convection *via* the blood flow as a central blood compartment. It is well known that anthropometric and demographic variables (age, sex, body-mass index) directly affect the heat characteristics of the human body (42), which explains why these variables have been found to independently predict hyperthermia during HIPEC (43, 44). The *active* system, in contrast, describes the thermoregulatory system. For example, common auto-regulatory responses to hyperthermia are vasodilatation and sweating, thereby increasing the heat redistribution to superficial areas of the body and the evaporative heat losses (**Figure 2**) (45). Ultimately, the computational models integrate these systems to predict temperature responses after heat or cold exposures in humans.

Only a few authors have approached HIPEC with mathematical or physical models of intra-abdominal hyperthermia. Examples include the mathematical human model proposed by Ladhari et al. and the animal treatment planning software model of Loke et al. (46, 47) Remarkably, these studies highlight the importance of patient and perfusion characteristics in the resultant intra-abdominal and core temperatures during HIPEC. Unfortunately, none of these models constitute a complete thermo-physical model, nor have they considered the effects of anesthesia in the thermoregulatory system (45, 48, 49).

**Figure 3:**
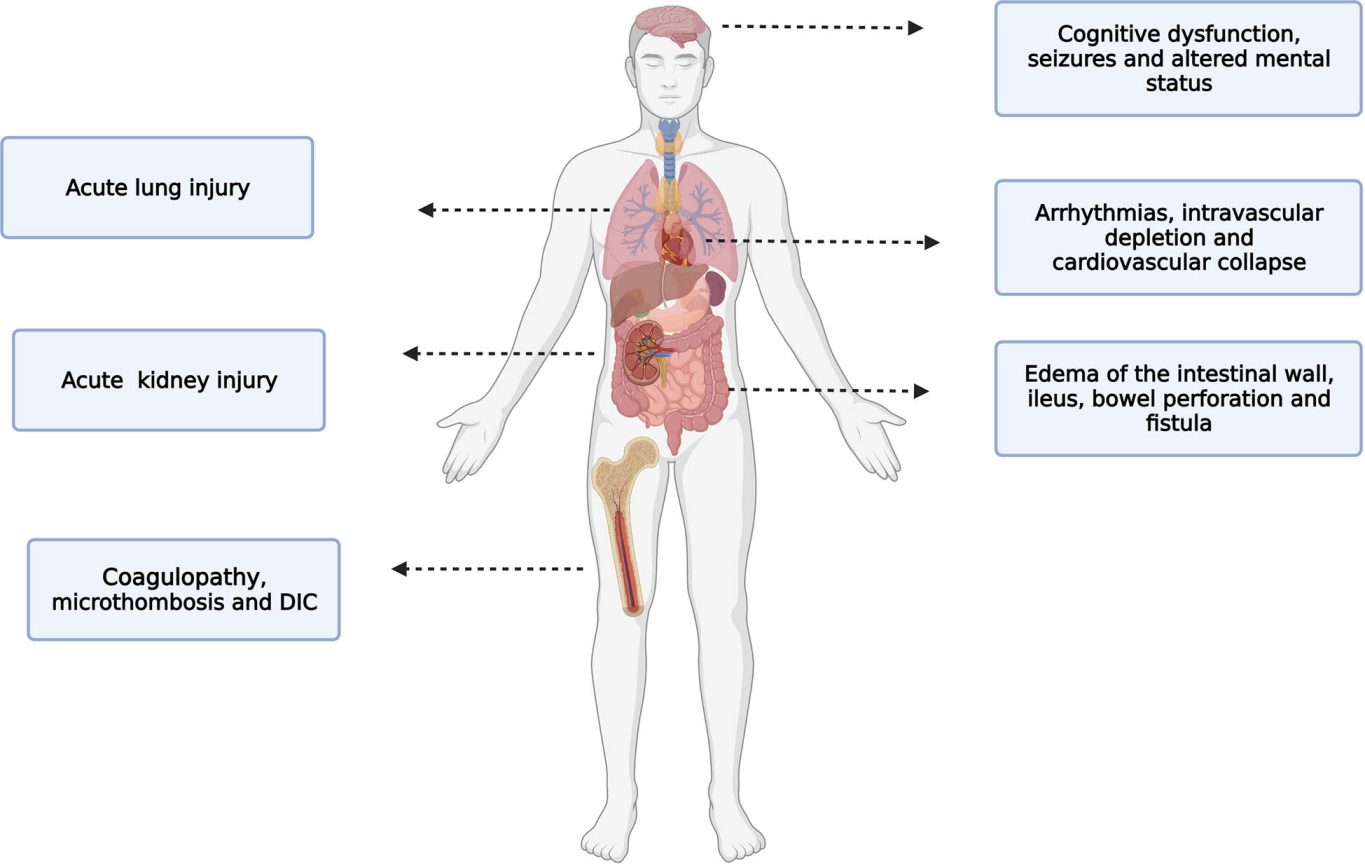
Side effects of hyperthermia. Side effects of hyperthermia during CRS-HIPEC.

The efficacy of CRS-HIPEC is directly related to the capacity to reach and maintain a target peritoneal temperature for as long as possible. However, with the continuous infusion of the heated perfusate, systemic hyperthermia is very likely to develop. While intra-abdominal hyperthermia may offer survival benefits, high core temperatures can lead to physiological derangements. Mild core (esophageal) hyperthermia is defined by core temperatures greater than 38°C, while moderate to severe hyperthermia begins at temperatures greater than 39°C. Patients undergoing CRS-HIPEC are at risk of moderate-to severe hyperthermia which is associated with several adverse effects (**Figure 3**) (50). These side effects are secondary to the close contact of the heated perfusate with the peritoneal cavity or are related to the systemic hyperthermia. In regard to direct effect of the heated perfusate, side effects include edema of the intestinal wall, ileus, bowel perforation, fistula and reduced cytotoxicity of some chemotherapeutics agents like mitomycin C (51). With regard to systemic hyperthermia, side effects include; cardiac arrhythmias, intravascular depletion, cardiovascular collapse, immunosuppression, poor neurologic outcomes, renal failure, coagulopathies, seizures and an increased risk of severe 30-day postoperative complications (44, 52–55). For instance, Hendrix et. al., found that patients undergoing CRS-HIPEC who reached severe hyperthermia (esophageal temperature of ≥39.5°C) at any time were more likely to develop postoperative complications (HR= 3.77, 95% CI 1.56-9,14), and this complication was most likely to be severe according to Clavien-Dindo classification (HR= 3.46, 95% CI 1.10-10.95) (44).

The central nervous system is particularly vulnerable to hyperthermia. Hyperthermia decreases cerebral perfusion when core temperature increases by more than 1.2°C (56). Patients who become acutely hyperthermic might experience cognitive dysfunction, seizures and change in consciousness (from lethargy to coma and death). Interestingly, hyperthermia can cause changes in memory even if the hyperthermic event is short (1 hour) and mild (body core temperature 38.8°C) (57, 58). Additionally, hyperthermia can also affect attention and processing information (59). A temperature above 40°C can be associated with a permanent neurological damage. This effects seems to be secondary to cellular changes and/or cell death. Other mechanism of central nervous system disarrangement includes direct neurotoxicity from hyperthermia combine with inflammation (60).

The circulatory response to hyperthermia is secondary to increased metabolism and increased oxygen demand (61). The hyperthermia induced hyper-metabolic state is characterized by an increase in heart rate, cardiac output, central venous pressure, systolic function, and a decreased in systemic vascular resistance (due to redistribution of blood flow to the cutaneous vasculature) and a decrease in circulating intravascular volume (62). Hyperthermia also effects the electrical activity of the heart by increasing the discharge of the sympathetic nervous system. This inotropic effect can lead to sinus tachycardia, junctional rhythm and sustained supraventricular and ventricular tachyarrhythmia (63). Additionally, the incremental activity of the sympathetic nervous system causes vasoconstriction of the splanchnic and renal circulation which combine with hypovolemia during HIPEC increases the risk of acute kidney injury. Interestingly, preclinical data suggest that hyperthermic perfusion itself does not aggravate HIPEC-induced acute renal failure and indeed is mostly the cytotoxic side effects of chemotherapy that causes the acute kidney injury in patients undergoing CRS-HIPEC (64).

It is important to point out that the majority of the side effects of hyperthermia has been described in preclinical and clinical models of hyperthermia such as sepsis, heat stroke or malignant hyperthermia, however the specific data regarding the side effect of hyperthermia in patients undergoing CRS-HIPEC remains unknown.

Several publications have described intra-abdominal and core-body temperature changes in patients undergoing closed HIPEC. In one study, Rettenmaier et al. collected data of the intra-abdominal fluid temperature in five locations: upper left and right quadrants, lower left and right quadrants, and the suprapubic region. HIPEC was administered with two inflow and two outflow catheters, using a flow rate of 1.6-1.8 L/min and aiming for an inflow-to-outflow gradient of 1.5°C. The authors found that the inflow-to-outflow gradient decreased significantly within the first 15 minutes of HIPEC and remained stable thereafter. The five regions demonstrated temperatures that followed such gradient, with minimal variation between them (19) Due to the proximity to the intra-abdominal fluid, the bladder temperatures also rose more rapidly in the initial period, and continued to show heat gain over time (43). Of note, the relationship between the perfusate and the bladder temperatures is likely to depend on the individual perfusate catheter configuration within the abdominal cavity (e.g., inflow placed in the upper or lower quadrants). These considerations are particularly relevant for closed HIPECs, given the inability to manipulate the catheter configuration once perfusion has started. Some authors have noted, a modest correlation between the change in the intraperitoneal and bladder temperatures and the change in core-body temperature (65). As such, bladder temperature changes may help clinicians guide changes in the cooling protocols to prevent unwanted systemic hyperthermia. Depending on the definition, the incidence of hyperthermia is quite variable with one third to one half of the patients experiencing it (43, 44). At the end of HIPEC, the abdominal cavity is drained of hyperthermic fluid and washed, allowing the patient to return to normothermic conditions. Hypothermia during this period is not uncommon and authors have reported the potentially devastating risks of rebound hypothermia and cardiac arrest after CRS- HIPEC (66).

The literature still needs to address several issues. First, it seems that intraperitoneal temperature stability is difficult to achieve despite established perfusion protocols (51). Exploring the potential causes of these problems (e.g., perfusion set up, patient’s position, type of device) may lead to a more predictable administration of therapeutic hyperthermia. Second, thermal dosimetry principles are difficult to apply to microscopic tumor spread throughout the peritoneal cavity and further research will help to improve the safety and efficacy of this medical intervention.

## Temperature management and cooling protocols during CRS-HIPEC

The HIPEC technique requires close communication between the surgical team, the perfusionist and the anesthesiologist. The role of the perfusionists is to control the temperature, the volume and the flow of the perfusate. The role of the anesthesiologist is to control body temperature necessary to maximize the effectiveness of intraperitoneal chemotherapy while avoiding adverse events associated with severe systemic hyperthermia. In an effort to avoid severe core hyperthermia, cooling protocols are widely employed during CRS-HIPEC. Unfortunately, current cooling protocols are not standardized and may involve the use of underbody cooling mattresses, ice packs around the head and axilla, and the use of forced air warmers operating at ambient room temperatures. A major disadvantage of these options is the inability to adequately cover major body surfaces and the lack of a constant closed feedback loop between the patient’s temperature and the cooling device. Therefore, there is not a constant re-adjustment of the cooling system and as a result, temperature control is unpredictable during chemoperfusion. Another technique widely used to avoid hyperthermia is to perform controlled hypothermia (by decreasing room temperature, cooling intravenous fluid and setting forced air warmers to ambient room temperature). The time for controlled hypothermia is not standardized. At our institution, it is around 30 minutes to 1 hour before the initiation of the HIPEC. The time for controlled hypothermia could be difficult to predict since the time required to achieve complete cytoreduction could be highly variable. Occasionally, severe systemic hyperthermia requires the reduction of intraperitoneal chemoperfusate temperature, potentially reducing its effectiveness.

There is relatively little data regarding temperature management during of CRS-HIPEC. For instance, the European Journal of Surgical Oncology published the Guidelines for Perioperative Care of Cytoreductive Surgery With or Without Hyperthermic Intraperitoneal Chemotherapy: Enhanced Recovery After Surgery (67). In this publication, the group of experts agreed to first; monitor patient’s temperature during CRS-HIPEC with esophageal temperature probe, second; keep patient normothermic (36 C°) during the cytoreduction phase, third; prevent hypothermia with forced air warmers and warming mattress, fourth; actively cool *via* forced air blowers on cool or ambient setting during HIPEC phase and fifth; allow an increase core body temperature to between 36-41C° during HIPEC phase. It should be pointed out (and as the authors mentioned on the guidelines) that while the strength of the data for active cooling and hypothermia is strong, the data regarding hyperthermia is limited and weak. The majority of the literature available describes the physiological implications of hyperthermia but none of the literature addresses or provides a more detailed guideline regarding temperature management during the hyperthermic phase of HIPEC. Several questions remain unanswered, such as what target temperature should the anesthesiologist achieve during controlled hypothermia before initiation of HIPEC? For how long does the patient need to be hypothermic before HIPEC? Does the timing of controlled hyporthermia change depending of the timing of chemopefusion? What is the target range of temperature during HIPEC? What is the maximum temperature allowed during HIPEC? How can we estimate which patients will have bigger delta changes in temperature? What about rebound hypothermia after HIPEC?

## Benefits of controlling temperature during CRS-HIPEC

### Survival

While several factors including gender, tumor histopathology, extra-abdominal disease, and the completeness of cytoreduction have been shown to influence survival in patients undergoing CRS-HIPEC, the role of intra-abdominal hyperthermia per se was not established until recently (68). A retrospective study of 214 patients undergoing CRS-HIPEC found that development of mild hyperthermia was associated with age and the type of chemotherapy. Prognostic factors associated with moderate to severe hyperthermia were the duration of the perfusion and blood transfusions. Interestingly, patients who were unable to achieve a bladder temperature of 38°C for 30 minutes during the perfusion had worsening recurrence free survival and overall survival (50) 9 361.

### Bowel function

In regards to temperature management of the perfusion, a retrospective study involving 59 patients found that patients who had stable temperature control (defined as change of temperature not exceeding 0.5C°) during the entire HIPEC had less pain, reduced time to flatus and shortened enteral nutrition and hospital stays. Unfortunately, oncological outcomes such as survival were not improved in the stable temperature group (51).

## Conclusion

In summary, the role of HIPEC is to maximize tumor cell death while minimizing systemic toxicity. Unfortunately there is no consensus regarding the optimal temperature necessary to reach the maximum benefit in regards to cancer prognosis while avoiding adverse events associated with severe local and systemic hyperthermia.

Despite protocols for cooling and warming during CRS-HIPEC, patients still develop episodes of severe hyperthermia and hypothermia. Overall, little is known about the impact of body temperature during CRS-HIPEC on oncological and perioperative outcomes. The amount of data published related to cooling protocols and cancer outcomes during HIPEC is still very limited. Standardization of temperature management and treatment during HIPEC will enhance the accuracy of scientific discussions. Ideally, data should be derived from a prospective randomized control study in which patients are kept on a constant target temperature for at least 30 minutes or more during the intraperitoneal chemotherapy. Continued research on this topic will allow HIPEC specialists to move from an empiric administration of hyperthermia to a thermophysical and evidence-based approach, which will promote the development of healthcare technologies and tools to improve the care of patients with peritoneal surface malignancies. Whether a tighter control of body core temperature during CRS-HPEC would promise improved outcomes remains unknown.

## Author contributions

MR, JG-L and CG-L: discussed ideas and prepared the manuscript. MR and JG-L: prepared figures and tables. PO-A and KF: improved manuscript and edited. All authors contributed to the article and approved the submitted version.
